# Editorial: Resistance to Salinity and Water Scarcity in Higher Plants. Insights From Extremophiles and Stress-Adapted Plants: Tools, Discoveries and Future Prospects

**DOI:** 10.3389/fpls.2019.00373

**Published:** 2019-04-02

**Authors:** Ruth Grene, Nicholas J. Provart, José M. Pardo

**Affiliations:** ^1^Department of Plant Pathology, Physiology, and Weed Science, College of Agriculture and Life Sciences, Virginia Tech, Blacksburg, VA, United States; ^2^Department of Cell and Systems Biology, University of Toronto, Toronto, ON, Canada; ^3^Instituto de Bioquímica Vegetal y Fotosíntesis, Seville, Spain

**Keywords:** salinity, water stress, resistance, adaptation, extremophiles

Abiotic stresses, such as excessive salinity and low water availability, have always presented major barriers to achieving high biomass and full yield potential in crops. Global climate change, with attendant increases in the severity of these abiotic stresses, will require directed adaptations of crop species on an unprecedented scale in order to sustain agricultural productivity. Current, and rapidly expanding, information on genome structure and function, primarily, but not exclusively, in model angiosperms, provides a starting point for a heightened understanding of genomic responses to drought and/or salt stress, within and across species. A crucial component that is leading to an acceleration in our understanding of stress responses is the ability to sequence whole genomes more rapidly and relatively inexpensively, and to begin to interpret the data that are accumulating, although such analyses are still very much in the beginning stages.

Superior stress tolerance may be due to the nature of defense-related protein-coding genes, expanded gene families of stress proteins, or the “stress-readiness” of tolerant species/ecotypes. This last mechanism—stress-readiness—may be due to stress-independent, constitutively higher expression of key “defense” genes through primed signaling networks or due to specific transcription factors, the role of post-transcriptional processes, such as alternative splicing, and combinations thereof (Haak et al., 2018). Lest this field fall into the trap of the “drunk looking under the streetlight for his keys” (that is, searching in easily studied areas), we must keep in mind that the discovery of novel mechanisms, novel gene family members, and novel signaling pathways is a distinct possibility as the combined analytical power of sequencing and data mining increases.

Filichkin et al. have conducted a comprehensive study, using both RNA-Seq and Iso-Seq, of alternative splicing events that occur in response to drought, heat and temperature stress in root, xylem and leaf tissue of poplar. They identified both stress-responsive isoforms that responded to each of the stresses that were tested, and isoforms that were unique to each of the three abiotic stresses. Interestingly, extensive stress-induced intron retention was detected in the data set, with the imposition of specific stresses being associated, in some cases, with an increase in fully spliced mRNAs, at the expense of isoforms that had retained introns, or the converse. Tissue specificity with respect to splicing events was also found. Isoforms encoding regulatory proteins showed differential intron retention (DIR), including some known poplar splicing factors. Additionally, co-regulation of DIR events occurred as a result of stress imposition. Non-coding mRNAs were also spliced, which brings the question of whether the differential splicing aims to enlarge the repertoire of cellular proteins or the physical presence of introns in the genome promotes survival under stress conditions by alternative mechanisms, as recently shown for the starvation response of the yeast *Saccharomyces cerevisiae* (Parenteau et al., 2019). Potentially, much more information lies in this large and very valuable dataset concerning the role of specific splice variants in known and novel stress response pathways. The data set is available to the public at Poplar Interactome GBrowse.

In another molecular study in this special topic, Mo et al. studied cassava (*Manihot esculenta*), which is an important staple food for around 800 million people. It is tolerant to environmental stresses such as drought and heat. Specifically, the authors cloned gene family members from the CBLs and CIPKs (calcineurin B-like proteins and CBL-interacting protein kinases, respectively, which are known to be involved in calcium-mediated stress signaling) and examined their expression patterns in response to different environmental stresses. The authors also tested whether pairs of the 8 CBLs and 25 CIPKs cloned for yeast two-hybrid analysis could interact in a yeast two-hybrid system. The authors also elegantly used a yeast-based system to reconstitute a functional SOS pathway (Quintero et al., 2002) and to show that co-expression of the cassava proteins SOS1, CBL10, and CIPK24 restored salt tolerance in transgenic yeast. These data recall previous findings in *A. thaliana* (Quan et al., 2007) and provide further evidence of the high degree of evolutionary conservation in stress-related signaling across plant species.

In the case of species that are closely related to mesophytes (plants that do not possess specific adaptation mechanisms for survival in extreme conditions) or ecotypes within species, their relatedness to better-studied close relatives increases the likelihood of discovering the genetic bases for their relatively superior stress resistance. Thus, these species or ecotypes present a rich potential resource for the identification of genes, processes, and pathways that confer this superior stress resistance. The coming together of more “classical” approaches such as QTL analysis with our increasing understanding of the genes and associated mechanisms that underlie stress responses in plants has made possible the identification of specific genes that contribute to stress tolerance, insights into details of known pathways, and, sometimes, the discovery of novel stress resistance phenomena. One approach to uncovering the mechanisms that underlie superior stress resistance lies in the study of these “extremophile” species, and ecotypes within species, that are adapted to thrive in more stressful environments than their near relatives.

Extremophiles have emerged in many plant families, often, in the case of crop plants, ecotypes or species that are closely related to less tolerant species of agronomic importance. The focus of Quan et al.'s  study was to show that wild rice, in this case, a salt-tolerant line of *Oryza rufipogon* Griff, could be used as a source to improve the salt tolerance of a cultivated variety of *O. sativa* ssp. Japonica, using genome resequencing of sensitive and tolerant lines, as well as tolerant hybrid offspring combined with QTL analysis to identify, where possible, the basis for increased tolerance. Since salt tolerance is a polygenic trait, much remains to be understood about its mechanistic basis. Nine QTLs for salt tolerance were identified by Quan et al. which included, for example, candidate genes such as *OsSKC1*, a sodium ion transporter, in addition to other ion transporters and many transcription factors whose role in salt tolerance is not yet well understood. Such data constitute a rich source for further exploration of the complexity of salt tolerance at the mechanistic level.

The work of Prusty et al. reveals an interesting, and perhaps unexpected mechanism, of “tissue tolerance” in some wild relatives of cultivated rice that exhibited relatively high salt tolerance. A tolerant tissue is defined as one which continues to function even in the presence of high levels of sodium, as opposed to sensitive tissues, which may co-exist in the same organism, which lack this capacity. Those cultivated rice cultivars that scored as salt tolerant were shown by Prusty et al. not to possess tissue tolerance, by this definition, whereas the tolerant wild rice species were tissue tolerant, specifically with respect to their shoots, but not their roots. The cells of the shoots of the wild rice species appeared to be able to sequester large amounts of sodium in their vacuoles, thus protecting the cytosol from toxic effects. This ability may be due to the action of specific members of the *NHX* gene family that evolved in wild rice, and were lost with cultivation, or to hitherto undiscovered mechanisms that bring about the sequestration of large amounts of sodium in the vacuole of the shoots of these wild rice genotypes. Another example of tissue tolerance is the extremophile xerophytic Asiatic shrub *Zygophyllum xanthoxylum*, studied by Xi et al. Like the salt tolerant wild rice studied by Prusty et al., this plant also accumulates sodium ion in its leaves, and in fact, under salt stress, tolerance increased, as measured by physiological traits, among them, expanded mesophyll cell size and increased ability to store water. More work is needed to pinpoint the genetic bases of the phenomenon of tissue tolerance, but these findings illustrate the possibility of discovering additional potential sources of salt tolerance.

Chen et al. also used a wild relative, in their case, of soybean, to identify potential regulators of alkali and salt stresses. This wild relative, *Glycine soja*, has the favorable trait of exhibiting superior tolerance to salt-alkali stress. The authors looked at 56 “response regulator” genes (GsRRs), which in previous studies were shown to be involved in diverse abiotic stresses. After grouping the genes into 5 subclasses, the authors examined transcriptional profiles of the genes in response to alkali stress and showed that the A1 and A2 subclass genes exhibit higher transcriptional levels than the B subclass genes. The response of GsRR2a in the A1 subclass was opposite for salt stress, however. In a nice use of Arabidopsis to test the function of the GsRRs, Chen et al. overexpressed GsRR2a in Arabidopsis and found that tolerance to alkali, but not salt, was increased.

Moving more into the genome-environment association field, Cortés and Blair used genotyping by sequencing of 86 geo-referenced wild accession of common bean, *Phaseolus vulgaris*, to discover single nucleotide polymorphisms. The authors then quantified allelic associations with a bioclimatic-based drought index under different models. For their optimum model, 115 SNPs in 90 regions across all 11 common bean chromosomes were associated with the bioclimatic-based drought index. A phototropic-responsive *NPH3* gene and a gene coding for an ankyrin repeat-containing protein were identified as potential drought tolerance candidates, but signatures of natural selection in these and other SNP-associated regions suggest that while drought tolerance might obviously be advantageous under drought conditions, it could be detrimental under humid conditions. It's complicated!

Last, Ulrike Bechtold from the University of Essex provides a mini-review on improving drought tolerance for this research topic. She points out that in spite of the identification of many desiccation inducible genes in dozens of studies in xerophytes or model species, there are few “gene-to-field” translational examples of genes that have resulted in improved crop performance under drought. She addresses the longstanding arguments between ecophysiologists who are studying drought tolerant extremophiles and molecular biologists who work with model species on how to “drought-proof” crops in the future. Also discussed are the different kinds of strategies (tolerance or avoidance) that plants use to survive drought and the occurrence of lineage-specific genes and non-coding RNAs in some of the extremophiles.

We feel that this collection of eight articles highlights a number of interesting avenues for exploration in the context of using natural variation and extremophiles to better understand how we can improve crops to be less thirsty and more tolerant of salt stress. Further improvements and price decreases in next generation sequencing technologies will enable more genome-environment kinds of studies with wild relatives of crop plants to be undertaken. Networks of interactions can be determined using other high throughput technologies. Better understanding of physiological responses will also play a key role.

## Author Contributions

All authors listed edited the manuscript, contributed to the Research Topic and wrote the Editorial.

### Conflict of Interest Statement

The authors declare that the research was conducted in the absence of any commercial or financial relationships that could be construed as a potential conflict of interest.
